# Nanocluster‐Assisted OH Ligand Modification Optimizes Activity and Stability of Fe‐N‐C Catalysts

**DOI:** 10.1002/advs.77615

**Published:** 2026-09-08

**Authors:** Xue Zhao, Junpeng Chen, Shice Wang, Zhao Yu, Jing Wang, Qiuming Peng, Ge Li

**Affiliations:** ^1^ State Key Laboratory of Metastable Materials Science and Technology Yanshan University Qinhuangdao People's Republic of China; ^2^ Department of Mechanical Engineering University of Alberta Edmonton Alberta Canada

**Keywords:** DFT, ligand modification, nanoclusters, oxygen reduction reaction, single atom catalysts

## Abstract

Achieving high activity and durability in platinum‐metal‐free electrocatalysts remains a challenge for proton‐exchange membrane fuel cells. Fe/N‐C catalysts are alternatives to platinum‐based catalysts, but their performance is limited by the instability of Fe‐N_x_ moieties under harsh operating conditions and site deactivation via uncontrolled ligand dynamics. Herein, we report a nanocluster‐assisted ligand‐anchoring strategy that establishes electronic coupling between Fe‐N_x_ sites and adjacent Fe nanoclusters, which serve as auxiliary anchors to stabilize OH ligands on atomic Fe centers. Morphological characterization and density‐functional‐theory calculations reveal that charge redistribution between the single‐atom site and neighboring clusters tailors the electronic environment of the Fe‐N_x_ reaction center. Ab initio molecular dynamics simulations confirm the dynamic stability of this cluster‐single‐atom motif with OH‐ligand modification under operating conditions. In situ Raman spectroscopy shows optimized evolution of oxygenated intermediates. The synergistic Fe single‐atom and nanocluster (Fe_SA+NC_‐NC) catalyst exhibits a half‐wave potential of 0.92 V, a kinetic current density of 183.57 mA cm^−2^ at 0.85 V, and retains 95% of its initial activity after 300 h of operation. The zinc‐air battery delivers a peak power density of 154 mW cm^−2^. This work presents a new paradigm for designing single‐atom catalysts by engineering the local coordination environment to overcome activity‐stability trade‐offs.

## Graphical abstract

A nanocluster‐assisted ligand engineering stabilizes OH‐modified FeN_4_ active sites through strong electronic coupling between isolated Fe atoms and adjacent nanoclusters. This synergy optimizes oxygen‐intermediate adsorption, lowers ORR energy barriers, and breaks the conventional activity–stability trade‐off, enabling high ORR activity, exceptional durability, and superior zinc–air battery performance.

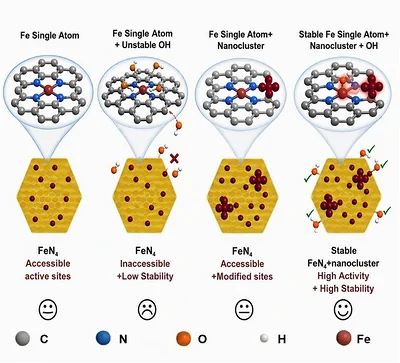

## 1 Introduction

The oxygen reduction reaction (ORR) is a key cathodic process in electrochemical energy conversion and storage technologies, including fuel cells and metal‐air batteries [1, 2]. However, the sluggish kinetics of the ORR severely hamper the efficiency and long‐term operation of these devices, necessitating the development of highly active and robust catalysts [3]. Among various alternatives to expensive platinum‐based materials, transition‐metal‐nitrogen‐carbon (M‐N‐C) catalysts have attracted particular attention, with Fe‐N‐C materials showing the most promising performance [4, 5, 6]. In these catalysts, atomically dispersed Fe‐N_x_ moieties are widely regarded as the primary active centers, offering remarkable activity by maximizing atomic utilization [7, 8, 9]. Despite this progress, Fe‐N‐C catalysts face inherent challenges that limit their practical application [10, 11, 12, 13, 14]. A critical bottleneck is the strong binding of oxygen intermediates, especially *OH, which is constrained by the universal scaling relationship [15, 16]. This strong adsorption impedes the desorption of intermediates and ultimately reduces the turnover frequency of the catalyst, a major obstacle in advancing ORR electrocatalysis.

To address these challenges, numerous strategies have been explored to tune the electronic environment of the active sites. Combining multiple active centers, for instance by coupling single metal atoms (M_SA_) with metal nanoclusters (M_NC_) or nanoparticles (M_NP_), has emerged as a particularly promising solution [17, 18, 19, 20, 21]. The synergistic sites created by the M_SA_+ M_NC_/ M_NP_ architecture can exhibit enhanced catalytic performance by integrating their distinct electronic and geometric properties, thereby optimizing the adsorption‐desorption energetics of reaction intermediates. Despite an explosive growth in research on M_SA_+ M_NC_/ M_NP_ synergistic catalysts in recent years, significant hurdles remain. These include the difficulty in achieving controllable synthesis of such complex structures and more critically deciphering the precise role each component plays in the overall catalytic process. To overcome these challenges, innovative designs and deeper mechanistic insights are essential.

**SCHEME 1:**
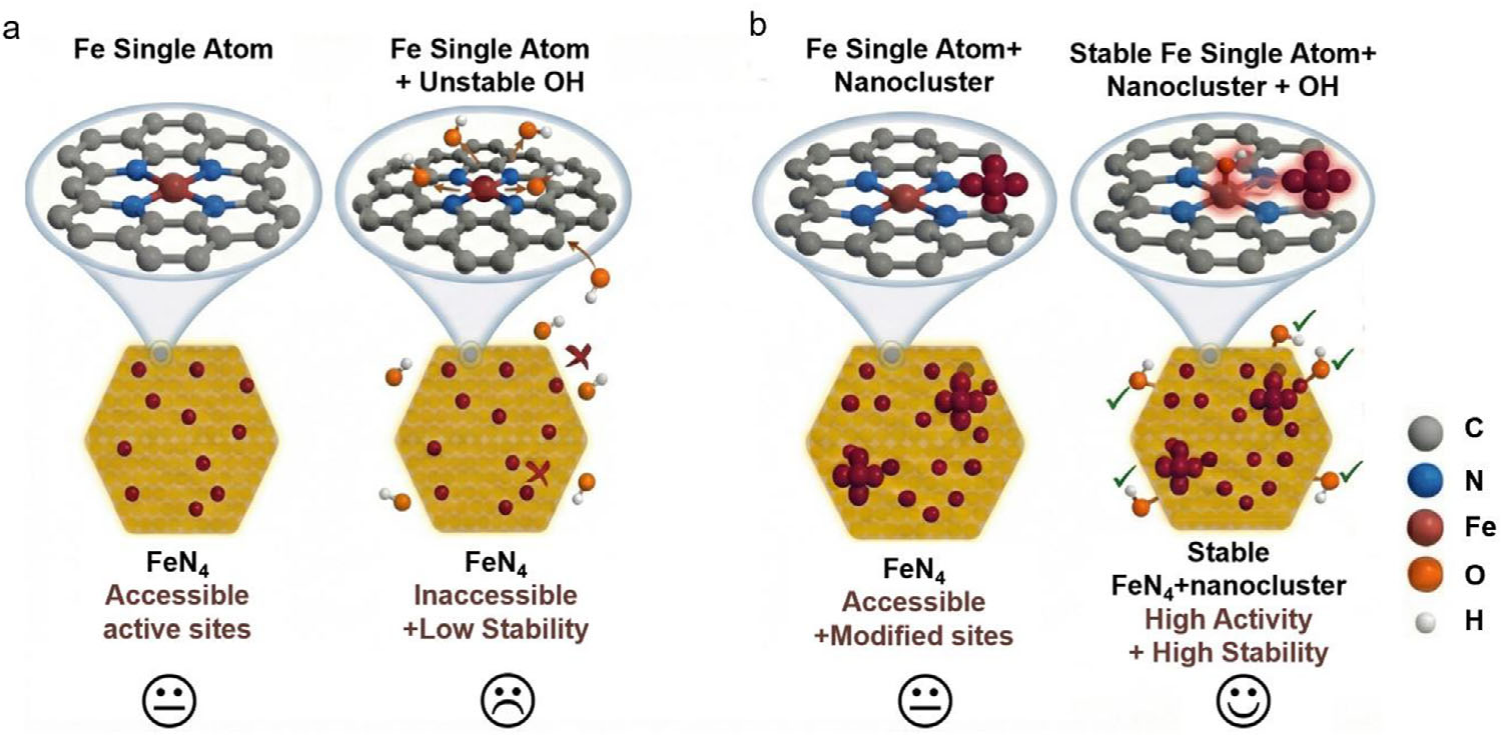
Schematic illustration of the stabilization mechanism for Fe single‐atom catalysts. (a) Conventional Fe single atoms (FeN_4_) suffer from instability and inaccessible active sites upon interaction with unstable OH species, leading to performance degradation. (b) The proposed strategy where neighboring nanoclusters induce stable OH ligand coordination on Fe single atoms. This modification modulates the local environment, resulting in robust FeN_4_ sites that exhibit simultaneously high activity and high stability.

Herein, we propose and demonstrate a novel neighboring nanocluster‐induced ligand modification strategy. As shown in Scheme 1, the presence of adjacent Fe nanoclusters regulates the local electronic structure of neighboring single‐atom sites, which stabilizes the in situ adsorption of an OH ligand on the Fe‐N_x_ moiety. This ligand modification directly optimizes the binding strength of the key *OH intermediate, thereby lowering the reaction energy barrier and boosting intrinsic activity. This crucial optimization effect was directly observed via in situ Raman spectroscopy, which captured the progressive accumulation of the OOH intermediate under operating conditions. Electrochemical measurements reveal that the spatially coupled Fe_SA+NC_‐NC catalyst achieves a half‐wave potential of 0.92 V and a kinetic current density of 183.57 mA cm^−2^ at 0.85 V, far exceeding Pt/C and catalysts containing only single atoms or nanoparticles. This superior electrochemical performance translated into excellent practical device application, where a zinc‐air battery (ZAB) equipped with the Fe_SA+NC_‐NC delivered a high peak power density of 154 mW cm^−2^ and exceptional long‐term cycling stability. Ab initio molecular dynamics (AIMD) simulations further verify that the OH‐modified coordination environment preserves structural integrity under both vacuum and aqueous conditions. The stable energy trajectories confirm that these active centers remain dynamically robust during operation. Long‐term durability tests further show that Fe_SA+NC_‐NC retains 95% of its activity after 300 h of continuous operation. By integrating systematic experiments with theoretical insights, we demonstrate that nanocluster‐induced ligand modification provides a practical pathway to break the conventional activity‐stability trade‐off in single‐atom catalysis.

## 2 Results and Discussion

### 2.1 Syntheses and Characteristics of Catalysts

**FIGURE 1:**
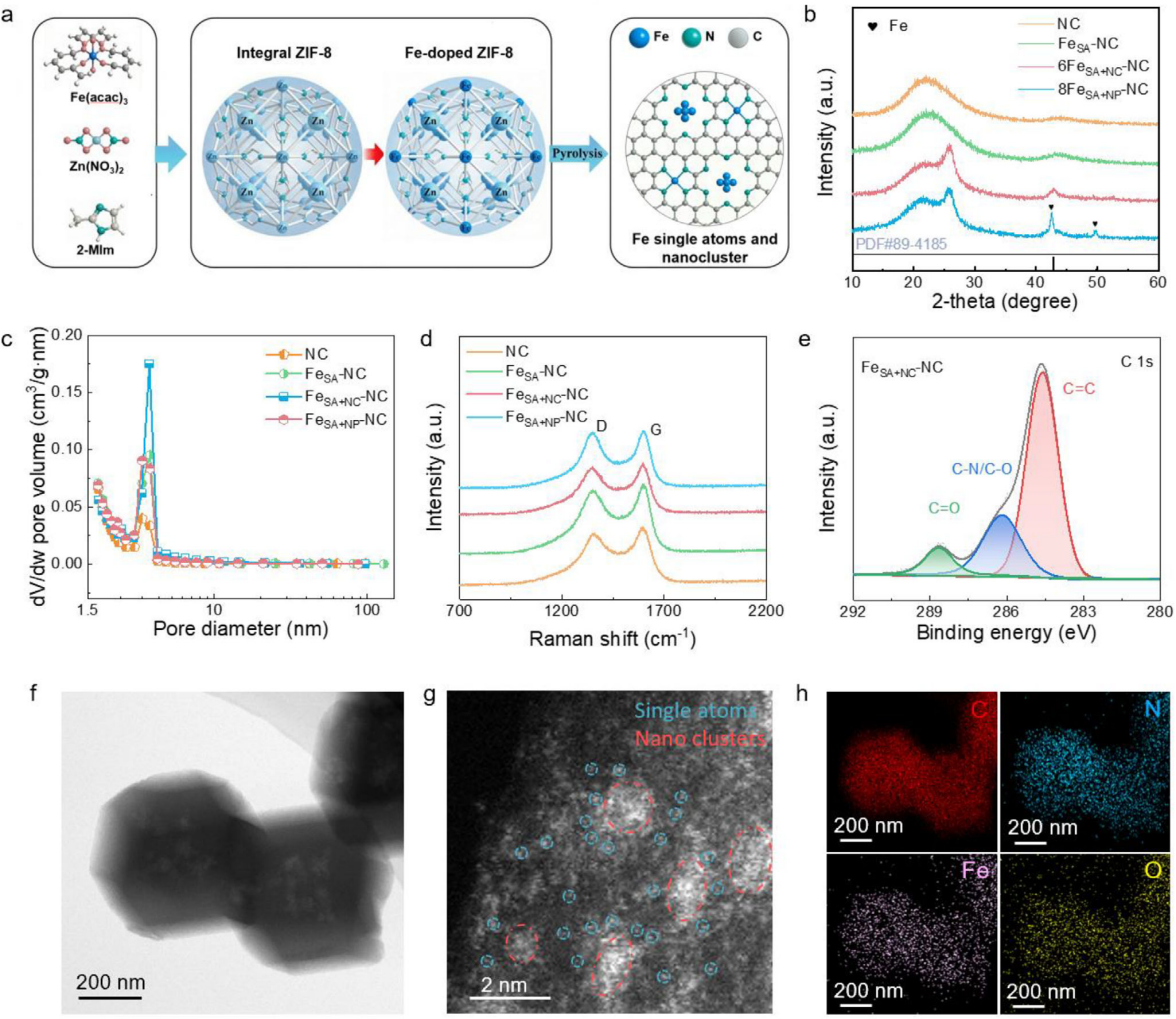
Structural design and morphology of the catalysts. (a) Schematic illustration of the synthesis process of catalysts. (b) XRD patterns of the prepared samples. Pore size distribution curves (c) and Raman spectra (d) of the catalysts. (e) High‐resolution C 1s XPS spectra of Fe_SA+NC_‐NC. TEM image (f) and AC‐HAADF‐STEM images (g) showing atomically dispersed Fe atoms and nanoclusters of Fe_SA+NC_‐NC. (h) Corresponding EDS elemental mapping images confirming the homogeneous distribution of C, N, O, and Fe.

The fabrication process of the nanocluster‐induced ligand‐modified Fe single‐atom catalysts is illustrated schematically in Figure 1a. The catalysts were synthesized by a one‐pot method using Fe(acac)_3_, Zn(NO_3_)_2_·6H_2_O, and 2‐methylimidazole in methanol. Specifically, Fe(acac)_3_ was added in amounts of 0.1, 0.2, and 0.4 g. The resulting samples after pyrolysis at 900°C under N_2_ were denoted as Fe_SA_‐NC (Fe single atoms), Fe_SA+NC_‐NC (Fe single atoms and nanoclusters), and Fe_SA+NP_‐NC (Fe single atoms and nanoparticles), respectively. A metal‐free control sample (NC) was also prepared under identical conditions without the addition of Fe(acac)_3_. Synthetic details are provided in the Methods section. The x‐ray diffraction (XRD) patterns of NC, Fe_SA_‐NC, Fe_SA+NC_‐NC, and Fe_SA+NP_‐NC are shown in Figure 1b. All samples exhibit a broad peak around 25.1°, attributed to the (002) plane of graphitic carbon [22], indicating that the carbon framework remains largely amorphous or disordered. For NC and Fe_SA_‐NC, no characteristic peaks of metallic Fe or iron oxides were observed, indicating that Fe atoms are atomically dispersed within the carbon matrix. The Fe_SA+NC_‐NC sample shows a weak and broad diffraction feature near 42.89°, which may correspond to the (111) plane of Fe nanoclusters [23, 24], implying partial aggregation of Fe species. In the case of Fe_SA+NP_‐NC, distinct diffraction peaks are observed at 42.89°and 49.95°, which can be assigned to the (111) and (200) planes of body‐centered cubic Fe [25], matching well with the standard card PDF#89‐4185, confirming the formation of Fe nanoparticles.

The Scanning electron microscopy (SEM) images (Figure S1) reveal that all samples retain the characteristic dodecahedral geometry, indicating good structural stability during pyrolysis. Nitrogen adsorption‐desorption measurements were further conducted to investigate the specific surface areas and pore structures of the samples. As summarized in Figure S2, the Brunauer‐Emmett‐Teller surface areas of NC, Fe_SA_‐NC, Fe_SA+NC_‐NC, and Fe_SA+NP_‐NC are 558.24, 603.21, 611.79, and 664.44 m^2^ g^−^^1^, respectively. The pore size distribution curves (Figure 1c) reveal that all samples possess a dominant mesoporous structure with a characteristic peak centered at approximately 3.5 nm. Notably, the Fe_SA+NC_‐NC sample exhibits the highest peak intensity compared to NC, Fe_SA_‐NC, and Fe_SA+NP_‐NC, indicating the most abundant mesopores and the largest pore volume. The relatively high surface area may facilitate exposure of active sites and promote efficient mass transfer during electrocatalytic reactions [26, 27]. The enhanced porosity is attributed to the catalytic effect of Fe species, which promote the thermal decomposition and pore evolution of the carbon framework during pyrolysis [28]. Raman spectra (Figure 1d) show the characteristic D (∼1350 cm^−^^1^) and G (∼1585–1600 cm^−^^1^) bands of sp^2^ carbon [29]. Compared with NC, the Fe‐containing samples exhibit higher I_D_/I_G_ ratios, indicating more defects and smaller graphitic domains introduced during pyrolysis [30]. Such defect‐rich carbon is beneficial for exposing active sites and improving charge/mass transport. To further investigate the surface chemical states of the carbon skeleton, x‐ray photoelectron spectroscopy (XPS) was performed. The high‐resolution C 1s spectra (Figure 1e) can be deconvoluted into three characteristic peaks centered at 284.8, 286.3, and 288.9 eV. The dominant peak at 284.8 eV is assigned to sp^2^ graphitic carbon (C═C), confirming the retention of a conductive graphitic framework which ensures efficient electron transfer. The other two peaks correspond to C─N/C─O and C═O species, respectively. The clear presence of C─N bonds verifies the successful incorporation of nitrogen heteroatoms into the carbon lattice.

Likewise, the Transmission electron microscopy (TEM) image further confirms the hollow interior and porous carbon shell structure, while no visible Fe aggregates are observed, suggesting the absence of large Fe particles. (Figure 1f). To investigate the atomic dispersion of Fe species, aberration‐corrected high‐angle annular dark‐field scanning transmission electron microscopy (AC‐HAADF‐STEM) was employed. As shown in Figure 1g, atomically dispersed Fe atoms are clearly observed along with sub‐nanometer Fe nanoclusters, indicating the coexistence of Fe single atoms and clusters within the carbon matrix. Notably, several Fe atoms are closely located near a small Fe cluster with an inter‐site distance below 0.5 nm, forming a spatially coupled Fe_SA+NC_ configuration. This proximity may facilitate synergistic electronic interaction between the single atoms and the nanoclusters, which is beneficial for catalytic performance [31]. Energy‐dispersive spectroscopy (EDS) elemental mapping (Figure 1h) confirms the homogeneous distribution of Fe, N, O, and C throughout the framework. It is also noted that a portion of Fe single atoms are spatially isolated from nanoclusters, which may function as conventional single‐atom active sites. In contrast, the Fe_SA_‐NC sample, prepared with lower Fe precursor content, shows only isolated bright dots in AC‐HAADF‐STEM images (Figure S3), corresponding to atomically dispersed Fe sites without detectable nanoclusters or particles. This result confirms the exclusive presence of Fe single atoms within the carbon matrix. On the other hand, the Fe_SA+NP_‐NC sample (with higher Fe content) displays a mixture of single atoms and aggregated Fe nanoparticles (Figure S4). Crystalline Fe nanoparticles are formed due to excessive Fe loading, potentially reducing atomic utilization efficiency.

**FIGURE 2:**
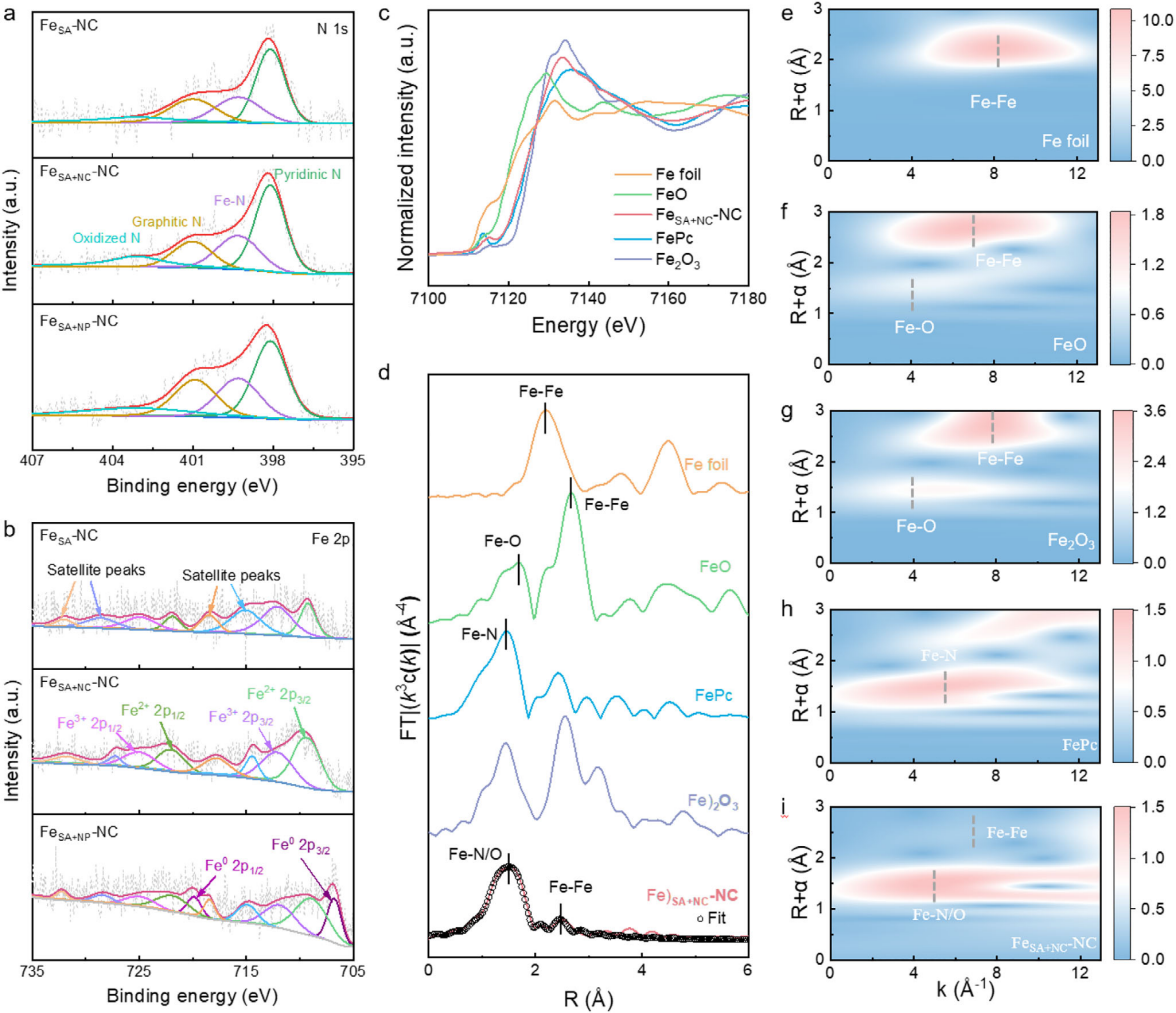
Electronic structure and coordination analysis of Fe species. (a) High‐resolution N 1s XPS spectra of Fe_SA_‐NC, Fe_SA+NC_‐NC and Fe_SA+NP_‐NC. (b) High‐resolution Fe 2p XPS spectra of the catalysts. (c) Normalized Fe K‐edge XANES spectra of Fe_SA+NC_‐NC compared with Fe foil, FeO, FePc and Fe_2_O_3_ references. (d) Fourier‐transformed EXAFS spectra at the Fe K‐edge. (e–i) WT‐EXAFS contour plots of Fe foil, FeO, Fe_2_O_3_, FePc, and Fe_SA+NC_‐NC.

The surface chemical states of the synthesized Fe_SA_‐NC, Fe_SA+NC_‐NC, and Fe_SA+NP_‐NC catalysts are examined using XPS. The survey spectra confirm the presence of C, O, N, and Fe elements in all samples (Figures S5 and S6). The elemental compositions are semiquantitatively analyzed by XPS (Table S1), and Fe content is further quantified by inductively coupled plasma optical emission spectroscopy (ICP‐OES, Table S2). The high‐resolution N 1s spectra of all samples exhibit characteristic peaks corresponding to pyridinic N (∼398.4 eV), Fe‐N coordination (∼399.3 eV), graphitic N (∼401.0 eV), and oxidized N species (∼403.1 eV), confirming the successful incorporation of nitrogen functionalities [32] (Figure 2a). Fe 2p spectra further elucidate the electronic states of Fe species (Figure 2b). All samples exhibit coexisting Fe^2+^ (∼710.6 eV) and Fe^3+^ (∼712.5 eV) [33]. Fe_SA_‐NC is dominated by Fe^3+^, consistent with isolated Fe‐N_x_ single‐atom configurations. In Fe_SA+NC_‐NC, both Fe^2+^ and Fe^3+^ signals are evident, supporting the presence of mixed Fe single atoms and nanoclusters. Notably, Fe_SA+NP_‐NC shows an additional shoulder at ∼707.2 eV, which corresponds to metallic Fe^0^, confirming the formation of Fe nanoparticles [34]. The relative increase of Fe^2+^ and the emergence of Fe° features indicate reduced coordination with nitrogen and a tendency toward particle aggregation at higher Fe loading.

The electronic structure and coordination environment of Fe species in the Fe_SA+NC_‐NC catalyst was systematically examined by X‐ray absorption spectroscopy (XAS). As shown in the normalized Fe K‐edge X‐ray Absorption Near Edge Structure (XANES) spectra (Figure 2c), the absorption edge of Fe_SA+NC_‐NC is located between those of Fe foil and Fe_2_O_3_, indicating that Fe is positively charged. The edge position is closest to that of FePc, implying that the dominant oxidation state is Fe^2+^, which can be attributed to atomically dispersed Fe‐N_x_ moieties. Meanwhile, the slight deviation toward lower energy suggests partial reduction of Fe, consistent with the presence of nanoclusters. The Fourier‐transformed Extended X‐ray Absorption Fine Structure (EXAFS) spectrum (Figure 2d) provides more structural information. A prominent peak at ∼1.44 Å corresponds to Fe‐N coordination, confirming the presence of isolated Fe single atoms. In addition, a weaker peak at ∼2.48 Å can be assigned to Fe‐Fe scattering, indicating that a fraction of Fe atoms aggregates into sub‐nanometer clusters. Fitting analysis yields coordination numbers of ∼4.0 for Fe‐N and ∼0.4 for Fe‐N‐Fe, quantitatively supporting the coexistence of well‐dispersed Fe‐N_x_ sites and small Fe clusters within the carbon matrix. Wavelet transform (WT) analysis (Figure 2e–i) further validates these findings. The Fe_SA+NC_‐NC sample exhibits a strong intensity maximum at ∼5 Å^−^^1^ in k‐space, associated with Fe–N backscattering, along with a secondary maximum at ∼6.5 Å^−^^1^, characteristic of Fe–Fe interactions. The simultaneous appearance of these features unambiguously demonstrates that both atomically dispersed Fe‐N_x_ moieties and neighboring Fe nanoclusters are present in the catalyst, forming spatially coupled configurations that are favorable for synergistic ORR catalysis.

### 2.2 Catalytic Performance

**FIGURE 3:**
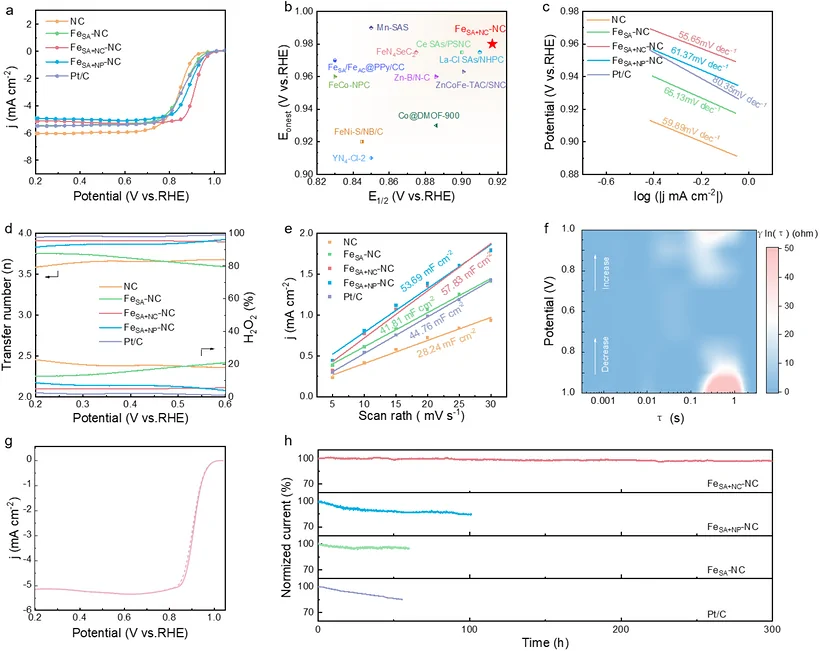
ORR performance of the Fe_SA+NC_‐NC catalyst. (a) LSV curves of NC, Fe_SA_‐NC, Fe_SA+NC_‐NC, Fe_SA+NP_‐NC and Pt/C in O_2_‐saturated 0.1 m KOH. (b) Comparison of E_onset_ and E_1/2_ with reported catalysts. (c) Tafel plots of the samples. (d) Electron transfer number and H_2_O_2_ yield from RRDE measurements. (e) C_dl_ values estimated from CV curves. (f) Corresponding contour plots of the calculated DRT results from Fe_SA+NC_‐NC. (g) Polarization curves before and after 10 000 cycles. (h) CA durability tests at a fixed potential for 300 h.

To comprehensively evaluate the bifunctional electrocatalytic properties of the prepared catalysts, we carried out rotating disk electrode measurements in alkaline media. The ORR activity of NC, Fe_SA_‐NC, Fe_SA+NC_‐NC, and Fe_SA+NP_‐NC catalysts was evaluated in O_2_‐saturated 0.1 m KOH solution. All electrode potentials were referenced to the reversible hydrogen electrode (vs. RHE). As shown in the cyclic voltammetry (CV) curves (Figure S7), all samples exhibit a distinct cathodic peak under O_2_‐saturated conditions, while no such peak is observed in Ar‐saturated electrolyte, confirming that the observed cathodic current originates from the ORR rather than intrinsic redox features of the carbon support. Linear sweep voltammetry (LSV) curves were recorded after subtracting the background currents measured under Ar atmosphere to eliminate capacitive and non‐faradaic contributions (Figure 3a). Among the tested catalysts, Fe_SA+NC_‐NC displays the most positive half‐wave potential (E_1/2_) of 0.920 V, outperforming Fe_SA+NP_‐NC (0.890 V), Fe_SA_‐NC (0.860 V), and NC (0.841 V), and previously reported nonprecious metal catalysts (Figure 3b and Table S3). The superior onset and half‐wave potentials highlight the enhanced intrinsic ORR activity enabled by the synergistic interaction between atomically dispersed Fe sites and adjacent nanoclusters. In addition, the limiting current density increases with rotation rate (400–2000 rpm, Figure S8), consistent with a diffusion‐controlled ORR process. The Tafel slope of Fe_SA+NC_‐NC is 55.65 mV dec^−1^ (Figure 3c), smaller than those of Fe_SA_‐NC (65.13 mV dec^−1^), Fe_SA+NP_‐NC (61.37 mV dec^−1^), and NC (59.89 mV dec^−1^), indicating accelerated electron transfer kinetics and reduced reaction barriers [35]. In addition, Fe_SA+NC_‐NC possesses a superior kinetic current density (j_k_) up to 183.57 mA cm^−2^ at 0.85 V, 27.7‐fold that of Pt/C (6.629 mA cm^−2^) (Figure S9). In contrast, Fe_SA_‐NC and NC display inferior ORR activity with reduced j_k_ (7.03 and 4.72 mA cm^−2^, respectively) and more negative E_1/2_ values, while Fe_SA+NP_‐NC shows only moderate activity (19.317 mA cm^−2^), demonstrating that the optimal intrinsic ORR activity of Fe_SA+NC_‐NC arises from a suitably modulated coordination environment via spatial coupling between Fe single‐atom sites and adjacent Fe nanoclusters. The ORR selectivity and reaction pathway were investigated by rotating ring‐disk electrode (RRDE) measurements (Figure 3d). Fe_SA+NC_‐NC exhibits an average electron transfer number (n) close to 4.0 and a H_2_O_2_ yield below 5% across the tested potential range, confirming a dominant 4‐electron reduction pathway and high selectivity toward H_2_O production. The electrochemically active surface area, estimated from the double‐layer capacitance (C_dl_) extracted via CV at different scan rates (Figure 3e and Figure S10), shows that Fe_SA+NC_‐NC has the largest C_dl_ value among all samples, implying the highest density of accessible electroactive sites [36]. To gain deeper insights into the interfacial reaction dynamics, in situ electrochemical impedance spectroscopy and distribution of relaxation times (DRT) analysis were employed. As shown in Figure S11, the dominant relaxation peak observed at ∼1.0 V is significantly suppressed as the potential increases, reflecting the rapid activation of the catalyst. The DRT heatmap (Figure 3f) further visualizes the evolution of polarization behaviors as a function of voltage, effectively decoupling the fast charge transfer steps (low τ) from the slower mass diffusion processes (high τ). The predominantly low intensity (blue region) across the voltage range during charge and discharge reveals that the Fe_SA+NC_‐NC catalyst maintains favorable reaction kinetics with minimized polarization resistance, further corroborating the accelerated electron transfer mechanism enabled by the coupled Fe dual‐sites.

The long‐term ORR stability of Fe_SA+NC_‐NC was evaluated through accelerated durability testing. After 10 000 potential cycles between 0.2 and 1.0 V vs. RHE (Figure 3g), its half‐wave potential shows a minimal negative shift from 0.920 to 0.915 V, demonstrating excellent electrochemical durability. Long‐term chronoamperometry (CA) at a fixed potential was performed for 300 h (Figure 3h). Fe_SA+NC_‐NC shows excellent durability, retaining ∼95% of its initial normalized current with negligible decay, whereas commercial Pt/C decays to ∼85%; Fe_SA_‐NC and Fe_SA+NP_‐NC display moderate losses, stabilizing at ∼87% and ∼85%, respectively. ICP‐OES monitoring indicates negligible Fe in the KOH electrolyte and negligible Fe loss from the catalyst, with the Fe content on Fe_SA+NC_‐NC mesh decreasing only from 1.21 to 1.18 wt% after cycling (Table S4). These results demonstrate that Fe_SA+NC_–NC is a promising ORR electrocatalyst with high activity and excellent long‐term stability.

### 2.3 In Situ Studies

**FIGURE 4:**
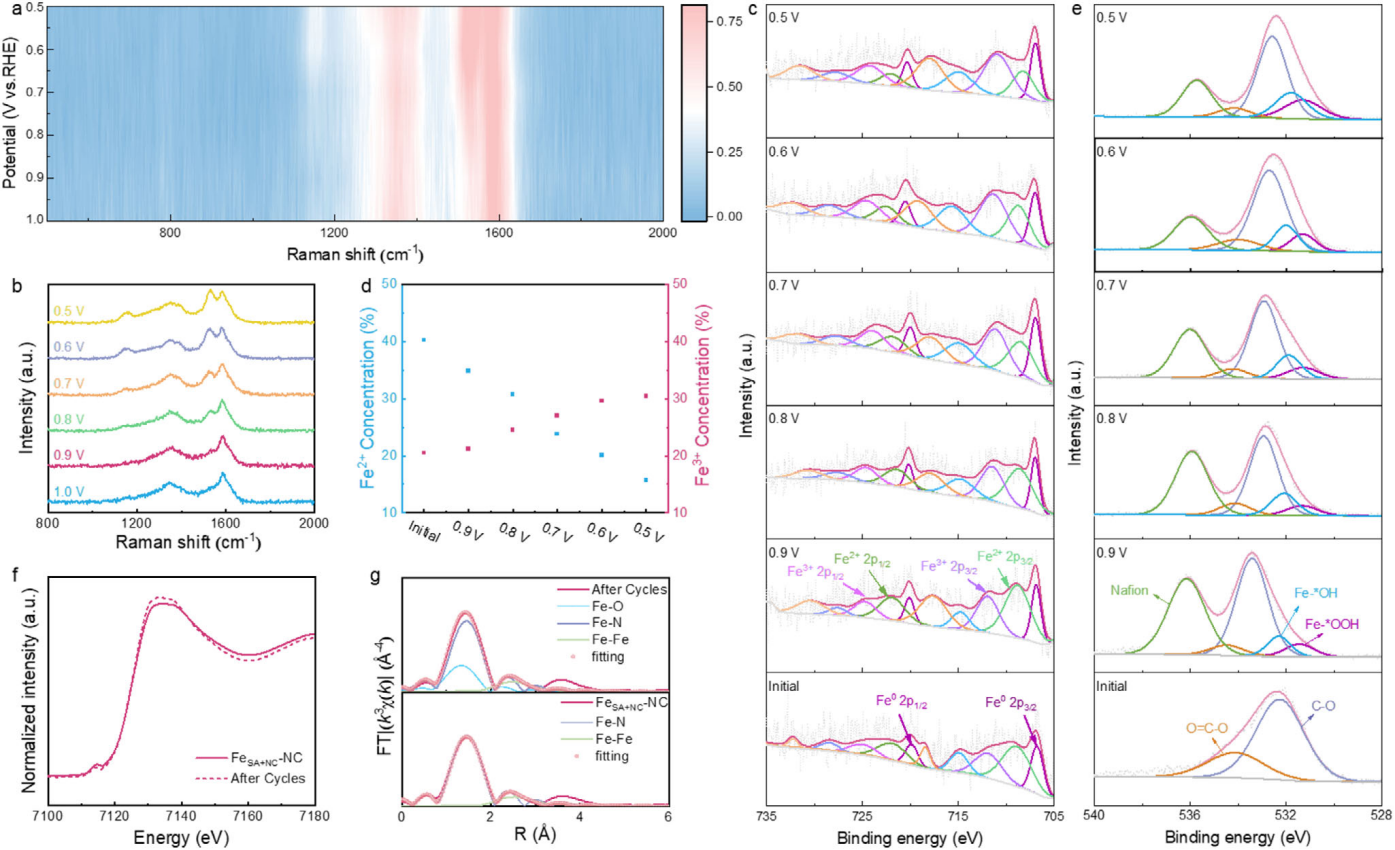
Operando and quasi‐operando spectroscopic evidence for potential‐dependent interfacial adsorption and Fe‐site electronic evolution during discharge. (a) Potential‐resolved in situ Raman intensity map collected from 1.0 to 0.5 V (vs. RHE). (b) In situ Raman spectra at selected potentials. (c) Fe 2p quasi‐operando XPS spectra and peak deconvolution at different potentials. (d) Quantified fractions of Fe^2^^+^ and Fe^3^^+^ as a function of discharge potential. (e) O 1s quasi‐operando XPS spectra and peak deconvolution showing the evolution of Fe–OH and Fe–OOH related components. (f) Fe K‐edge XANES spectra before and after cycling. (g) Fe K‐edge FT‐EXAFS spectra before and after cycling.

To elucidate the atomic and electronic structure of the Fe_SA+NC_‐NC catalyst under operational conditions, we performed in situ Raman spectroscopy during the ORR in an O_2_‐saturated 0.1 m KOH solution (Figure 4a,b). For the Fe_SA+NC_‐NC sample, the spectra prominently feature peaks at approximately 1590 and 1342 cm^−1^, which are attributed to the characteristic G and D bands of the carbon support, respectively. More importantly, as the applied potential was decreased, new peaks emerged and intensified at approximately 1128 and 1527 cm^−1^. These are assigned to the vibrational modes of the *O_2_^−^ and *OOH intermediates, respectively. The intensity of the *OOH peak, in particular, grew significantly as the potential was swept from 1.0 V down to 0.5 V. This observation indicates the progressive formation and accumulation of *OOH on the active sites during the ORR. As a comparison, in situ Raman spectroscopy was also conducted on the Fe_SA_‐NC catalyst under identical conditions (Figure S12). In contrast to Fe_SA+NC_‐NC, no obvious potential‐dependent evolution of the *O_2_^−^‐ and *OOH‐related Raman bands was observed for Fe_SA_‐NC, suggesting its relatively limited capability for oxygen activation and intermediate formation. These results indicate that the integration of neighboring Fe nanoclusters promotes the formation of oxygenated ORR intermediates on the FeN_x_ active sites, which is consistent with the superior electrochemical activity of Fe_SA+NC_‐NC. Therefore, the neighboring Fe nanoclusters facilitate electronic modulation of the FeN_x_ sites, optimize the adsorption behavior of oxygenated intermediates, and consequently enhance the ORR performance.

Quasi operando XPS was employed to quantify the potential dependent evolution of the electronic state of Fe sites during ORR discharge. The Fe 2p spectra at a series of discharge potentials are shown in Figure 4c and can be deconvoluted into Fe^2+^ and Fe^3+^ components, with the corresponding composition trends summarized in Figure 4d. Upon lowering the potential, the apparent valence distribution shifts monotonically toward a higher oxidation state. The Fe^3+^ fraction increases from about 20 percent to about 33 to 35 percent, while the Fe^2+^ fraction decreases from about 40 percent to about 15 percent as the potential approaches 0.5 V. This continuous redistribution indicates that the Fe‐N_x_ reaction centers become progressively more electron deficient under discharge, consistent with an adsorption coupled electronic modulation rather than a static active site picture. In parallel, the O 1s spectra in Figure 4e reveal strengthened contributions that are assignable to Fe‐OOH and Fe‐OH species as the electrode is discharged. The Fe‐OOH related signal evidences the formation of an oxygenated intermediate associated with O_2_ activation and early proton coupled electron transfer steps on Fe centers. With further decrease in potential, the Fe‐OH contribution becomes increasingly dominant and reaches its maximum prominence at 0.5 V, indicating a substantially higher population or longer residence of OH derived ligation on the Fe single atom sites at low potential. Importantly, the simultaneous increase of Fe^3+^ character in Fe 2p and the growth of Fe‐OH and Fe‐OOH signals in O 1s support a coupled process in which oxygenated ligands withdraw electron density from the atomic Fe center. When interpreted together with the cluster assisted ligand anchoring concept proposed in this work, these quasi‐operando trends are consistent with a scenario where adjacent Fe nanoclusters electronically couple with Fe‐N_x_ single atom sites and stabilize OH ligation on the atomic Fe centers, thereby regulating the intermediate adsorption landscape under realistic discharge conditions.

To further clarify the role of neighboring Fe nanoclusters in the proposed ligand‐anchoring mechanism, quasi‐operando XPS measurements were also performed on the Fe_SA_‐NC catalyst as a control sample (Figure S13). As shown in Figure S13a, the Fe 2p spectra exhibit only moderate potential‐dependent evolution during ORR discharge. Although a gradual redistribution between Fe^2+^ and Fe^3+^ species is observed upon decreasing the potential, the extent of valence variation is noticeably weaker than that observed for Fe_SA+NC_‐NC, indicating relatively limited adsorption‐coupled electronic modulation of isolated Fe‐N_x_ sites. Meanwhile, the O 1s spectra (Figure S13b) reveal the formation of both Fe‐OH and Fe‐OOH species during discharge. However, compared with Fe_SA+NC_‐NC, the relative areas of the Fe‐OH and Fe‐OOH components remain substantially smaller, particularly for the Fe‐OH contribution. These results suggest that isolated Fe‐N_x_ sites alone exhibit a lower population of oxygenated ligands under operating conditions. In contrast, neighboring Fe nanoclusters significantly enhance the electronic interaction between Fe‐N_x_ sites and adsorbed oxygen species, promoting the retention of OH ligands on atomic Fe centers. Together with the quasi‐operando observations for Fe_SA+NC_‐NC, these findings provide further support for the proposed nanocluster‐assisted ligand‐anchoring mechanism.

Synchrotron XAS was performed to examine whether the local electronic and coordination structure of Fe sites is preserved after the durability test. In Figure 4f, the Fe K‐edge XANES of the cycled electrode shows a small shift of the absorption edge toward higher energy while largely maintaining the overall spectral profile, indicating a modest increase in the average Fe oxidation state after operation. This trend is consistent with the persistence, or partial enrichment, of oxygenated ligation at the Fe center under working conditions, in agreement with the post operation surface analyses. In addition, the Raman spectra collected before and after durability cycling show largely retained D and G bands of the carbon framework (Figure S14), suggesting that no severe carbon corrosion or structural collapse occurs during long‐term operation. The FT‐EXAFS results further support the structural integrity at the atomic‐site level. As shown in Figure 4g, the spectra are dominated by the first‐shell peak and do not exhibit an evident growth of Fe‐Fe scattering that would be characteristic of metallic aggregation. Quantitative fitting at the Fe K‐edge in Table S5 indicates that the primary Fe‐N coordination is essentially unchanged after cycling, with a coordination number of 4.0 ± 0.1 and an average Fe‐N distance of 2.00 ± 0.01 Å for both the fresh and cycled samples, together with a similar Debye‐Waller factor of 0.007 Å^2^. In contrast, an additional Fe‐O contribution becomes resolvable after cycling, with a coordination number of 1.0 ± 0.1 at 1.85 ± 0.01 Å, directly evidencing oxygen ligation developed or retained during durability operation. Meanwhile, the higher‐shell Fe‐related path remains minor and does not increase, with a coordination number of 0.4 ± 0.1 at about 3.00 Å before and after cycling, while the associated disorder slightly increases, consistent with subtle local rearrangement rather than particle‐level growth. The low fitting residuals further validate the reliability of the extracted parameters, with R‐factors of 0.0022 for the fresh sample and 0.0004 after cycling. Collectively, Figure 4f,g, together with Table S5, indicate that durability cycling mainly induces site‐scale electronic and coordination reorganization involving oxygenated ligation, while the Fe species remain predominantly atomically dispersed without pronounced Fe‐Fe aggregation.

### 2.4 Theoretical Analysis

**FIGURE 5:**
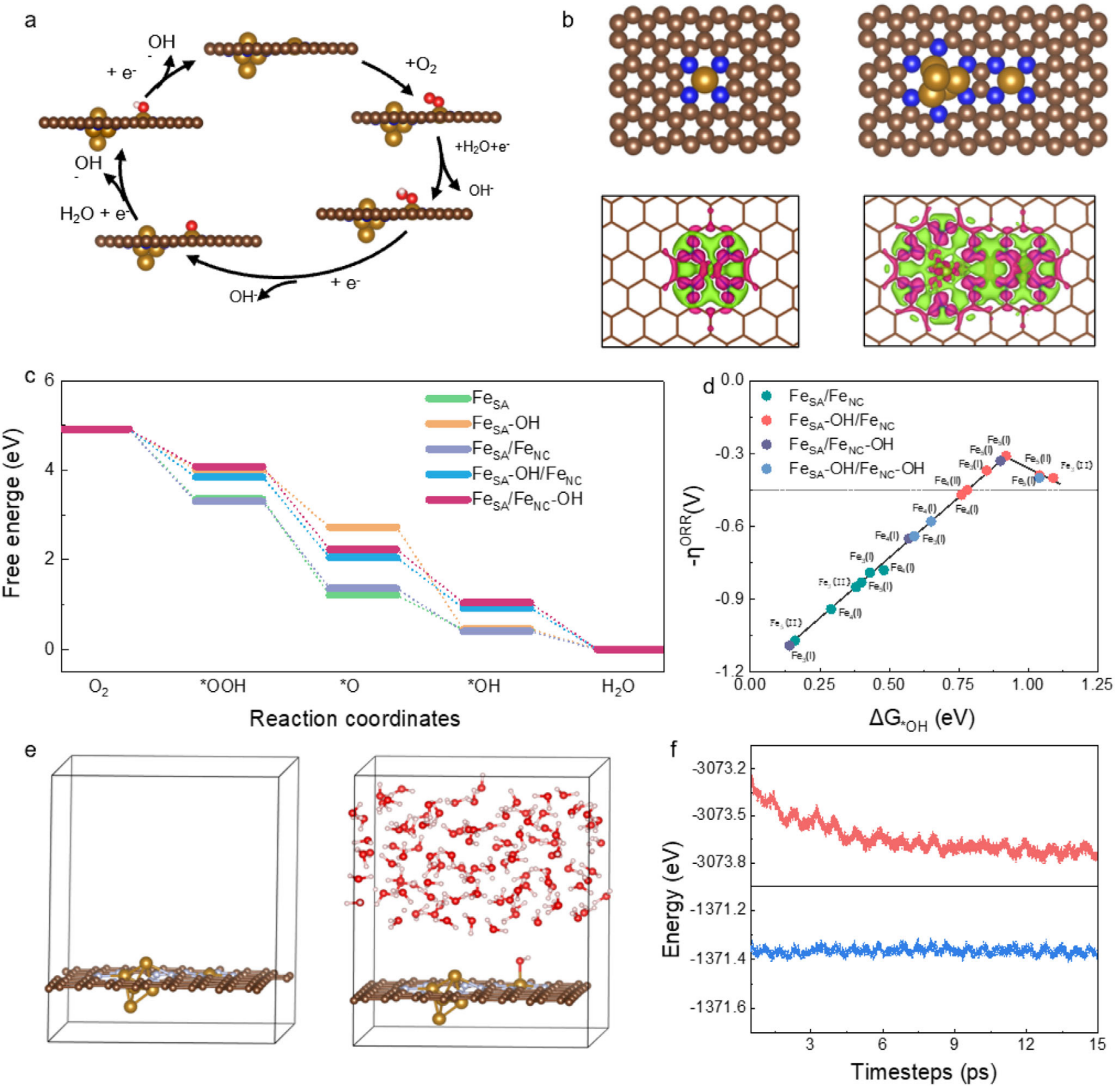
Theoretical calculations and simulations of the catalysts. (a) Proposed 4e^−^ ORR pathway involving O_2_, *OOH, *O, and OH intermediates. (b) Structural models and corresponding charge‐density‐difference maps of Fe_SA_ and Fe_SA_/Fe_NC_. (c) Free energy diagrams of the ORR pathway at 1.23 V for different models. (d) Calculated catalytic activity volcano plots. (e) AIMD simulation models of Fe_SA_/Fe_NC_ in vacuum and in an explicit aqueous environment. (f) Total energy variations during AIMD simulations.

Theoretical calculations were performed to elucidate the reaction mechanism on Fe single atoms and the synergistic effect arising from adjacent Fe nanoclusters anchored on N‐doped carbon. The elementary intermediates and their interconversion along the 4‐electron ORR pathway (*OOH → *O → *OH → H_2_O) are summarized in Figure 5a [37]. Specifically, we constructed Fe_SA_/Fe_NC_ hetero‐dimers composed of one Fe‐N_x_ site and a neighboring Fe_x_N_6_ cluster (x = 3, 4, 5) embedded in N‐doped carbon. Two geometries were considered: Fe_SA_/Fe_NC_(I) and Fe_SA_/Fe_NC_(II), with Fe‐center separations of 6.21 and 7.49 Å, respectively (Figures S15–S17). Upon relaxation, Fe_3_N_6_ forms a planar triangular Fe_3_ unit; Fe_4_N_6_ adds an apical Fe to yield a trigonal‐pyramidal motif; Fe_5_N_6_ adds the opposite apical Fe, approaching an octahedral configuration. Charge‐density‐difference maps display pronounced electron accumulation/depletion around both the Fe‐N_x_ unit and the neighboring Fe cluster, indicating strong interaction via electron exchange (Figure 5b). These results indicate that the integration of nanoclusters led to electron [38]. The corresponding free‐energy diagrams calculated at 1.23 V are presented in Figure 5c and Figures S18–S20. All free‐energy profiles show a monotonic downhill ORR at Fe single‐atom sites. The process is thermodynamically favorable. For isolated Fe‐N_x_, the rate‐limiting step of ORR is *OH desorption with an overpotential of 0.79 V, and this remains essentially unchanged even in the OH modified environment. Due to the modulated electronic environment, the introduction of a neighboring Fe nanocluster significantly enhances the stability of the OH ligand on the Fe single‐atom site, making this configuration more favorable and robust compared to an isolated Fe‐N_x_ site. This stably adsorbed OH ligand modulates the electronic structure of the Fe center, which in turn optimizes the binding strength of the key *OH reaction intermediate. This directly addresses the overly strong adsorption identified as the rate‐limiting factor, thereby lowering the overall reaction energy barrier. As a result, the overpotential decreases to 0.31 V on the OH ligated Fe‐N_x_ site, where the rate limiting step (RLS) remains *OH desorption, and to 0.39 V on the OH modified Fe nanocluster, where the RLS shifts to *OOH desorption. Importantly, regardless of whether OH ligand adsorbs on the single‐atom site or the nanocluster, the presence of the Fe cluster markedly optimizes the catalytic activity. Meanwhile, structural relaxations show that in all configurations the Fe site binds O_2_ in an end‐on mode. The O─O and Fe─O bond lengths exhibit a clear inverse correlation. In the unmodified Fe_SA_/Fe_NC_, O_2_ is overbound with progressive shortening of Fe─O and pronounced elongation of O─O. Hydroxylation markedly reduces the O_2_ adsorption energy at Fe_SA_. Compared with the unmodified system, the O─O bond length increases by 0.01 Å and the Fe─O bond length increases by 0.13 Å, indicating that the OH ligand weakens adsorption at Fe_SA_ and enhances catalytic performance (Table S6). Moreover, the calculated OH binding energies further support the proposed nanocluster‐assisted ligand‐anchoring mechanism. As summarized in Table S7, the OH binding energy changes from 0.44 eV for Fe_SA_‐NC to 0.40 eV in the presence of neighboring Fe nanoclusters. This result suggests that adjacent Fe nanoclusters modify the interaction between OH ligands and Fe‐N_x_ sites through electronic coupling, which is consistent with the experimentally observed enhancement of Fe‐OH populations under operating conditions. Fe 3d projected density of states analyses were further conducted to clarify the electronic modulation of the Fe active center (Figure S21). The Fe 3d states near the Fermi level are clearly redistributed after introducing neighboring Fe nanoclusters and OH modification, confirming that the coupled Fe cluster and OH ligand jointly regulate the electronic structure of the FeN_x_ site and thereby optimize the adsorption behavior of ORR intermediates.

To further clarify the role of OH modification, the volcano plot in Figure 5d illustrates the relationship between the ORR overpotential and the *OH adsorption free energy (ΔG*OH). The unmodified (Fe_SA_/Fe_NC_, teal dots) and nanocluster‐OH modified (Fe_SA_/Fe_NC_‐OH, purple dots) catalysts are predominantly located on the left side of the volcano. This position signifies an overly strong binding of *OH intermediates, which impedes their desorption and results in suboptimal ORR activity. Conversely, catalysts with a hydroxyl‐modified single‐atom site (Fe_SA_‐OH/Fe_NC_, red dots) are concentrated near the apex of the volcano. This peak region represents a moderate and more optimal *OH binding strength, which lowers the reaction energy barrier and leads to enhanced catalytic performance [39]. Notably, the configuration with an OH ligand on the Fe‐N_x_ single‐atom site resides at the pinnacle of the volcano, achieving the lowest overpotential of 0.31 V. This demonstrates that rationally engineering the active site with an OH‐ligand is an effective strategy to tune electronic environment of the catalysts for optimal ORR activity.

To further verify the stability of the optimized active sites, AIMD simulations were performed. As shown in Figure 5e, the Fe_SA_/Fe_NC_ model was constructed both in vacuum and in explicit aqueous environments to mimic the real electrochemical conditions. In the presence of water molecules, the Fe‐N_x_ and neighboring Fe nanocluster sites retained their structural integrity without obvious distortion, indicating that the OH‐modified coordination environment is dynamically stable. The corresponding energy trajectories in Figure 5f further confirm this stability. While fluctuations are observed, the total energy gradually converges and remains steady throughout the simulation, demonstrating that the Fe_SA_/Fe_NC_ active centers are not only thermodynamically favorable for ORR but also robust under aqueous environments.

### 2.5 Battery Performance

**FIGURE 6:**
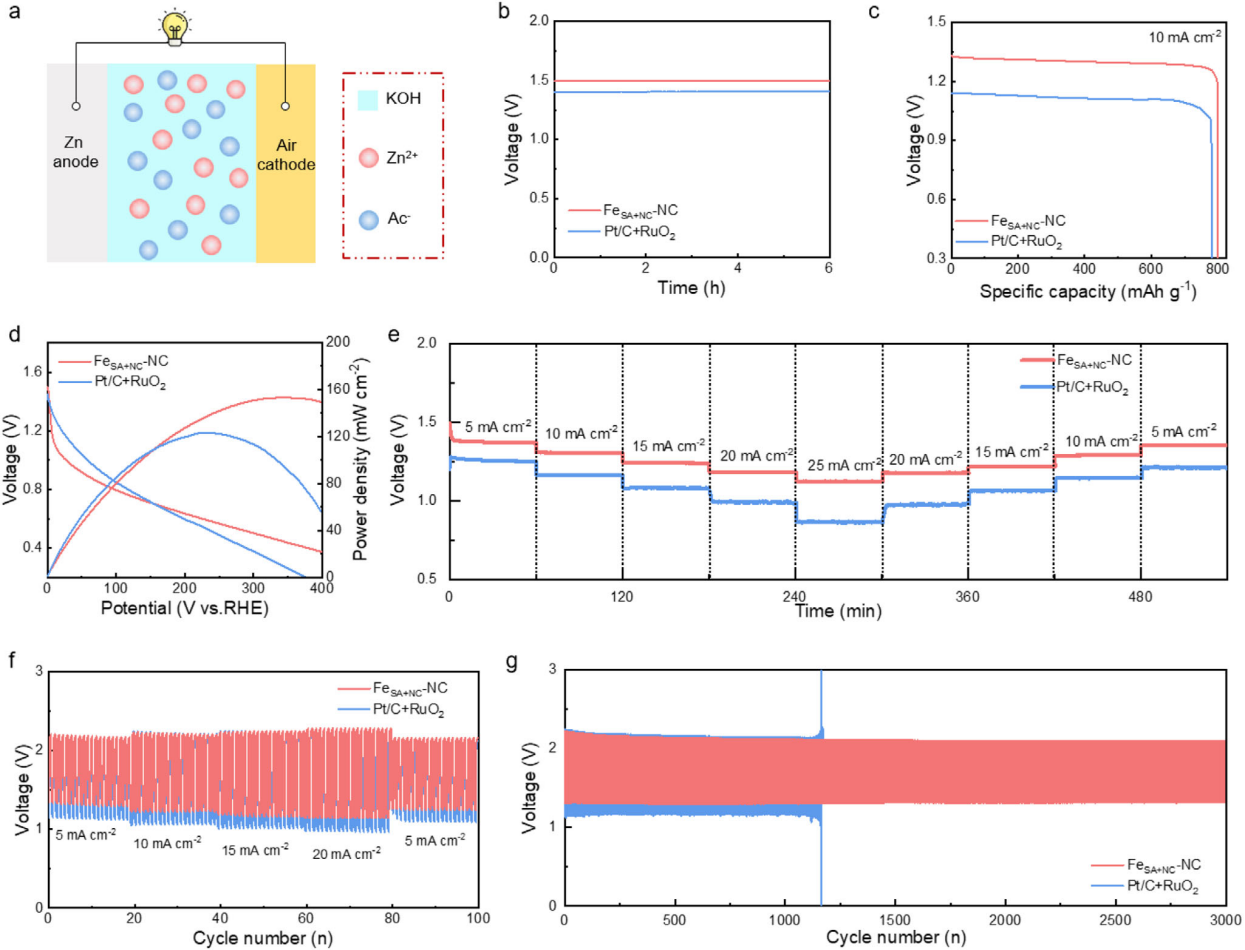
Aqueous ZAB performance with Fe_SA+NC_‐NC air cathode in comparison with noble metal of 40% Pt/C. (a) Schematic illustration of the ZAB setup. (b) Open circuit voltage curves. (c) Specific capacity curves at a current density of 10 mA cm^−2^. (d) Discharge polarization curves and corresponding areal power density curves. (e) Discharge curves at different current densities. (f) The galvanostatic charge‐discharge curves at room temperature with various current densities. (g) Long‐term cyclic stability tests at a current density of 5 mA cm^−2^.

To examine the viability of the Fe_SA+NC_‐NC catalyst in a practical application, we assembled and tested a primary ZAB featuring an air cathode loaded with the Fe_SA+NC_‐NC catalyst, as schematically illustrated in Figure 6a. The assembled ZAB demonstrated a high open‐circuit voltage (OCV) of 1.487 V, which is notably superior to the 1.404 V recorded for the benchmark ZAB constructed with a commercial Pt/C catalyst (Figure 6b). To further quantify the energy capacity, galvanostatic discharge measurements were conducted at a current density of 5 mA cm^−2^ (Figure 6c). The Fe_SA+NC_‐NC driven ZAB delivered a high specific capacity of 792 mAh g^−1^, surpassing the Pt/C+RuO_2_ based battery and indicating higher utilization efficiency of the zinc anode. The discharge performance was thoroughly evaluated through polarization and power density plots (Figure 6d). The ZAB equipped with the Fe_SA+NC_‐NC cathode consistently delivered higher discharge currents across the voltage range compared to the Pt/C counterpart. This superior performance culminated in a remarkable peak power density of 154 mW cm^−2^, significantly outperforming the 123 mW cm^−2^ achieved by the ZAB using the commercial Pt/C cathode.

The rate capability and mass transport properties were subsequently investigated. Figure 6e displays the galvanostatic discharge curves at various current densities ranging from 5 to 25 mA cm^−2^ The Fe_SA+NC_‐NC cathode maintained stable and high voltage plateaus even at a high current density of 25 mA cm^−2^, demonstrating excellent reaction kinetics. To probe the rechargeability under fluctuating power demands, the batteries were subjected to a galvanostatic charge‐discharge rate test (Figure 6f). As the current density increased from 5 to 20 mA cm^−2^, the ZAB with Fe_SA+NC_‐NC displayed a stable, step‐like voltage response with narrow voltage gaps. Crucially, it maintained a lower overpotential than the Pt/C‐based ZAB across all current densities. Finally, the long‐term durability was assessed via galvanostatic charge‐discharge cycling at 5 mA cm^−2^ (Figure 6g). The device exhibited exceptional stability with negligible voltage degradation even after 3000 continuous cycles (over 500 h), highlighting the promising potential of the Fe_SA+NC_‐NC catalyst for large‐scale, high‐performance energy applications. Moreover, the ZAB was further evaluated under a higher current density of 10 mA cm^−2^ to assess its durability under more demanding operating conditions (Figure S22). The Fe_SA+NC_‐NC‐based ZAB maintained a stable charge‐discharge voltage profile for 300 h, whereas the Pt/C+RuO_2_‐based ZAB showed pronounced voltage fluctuation and rapid degradation after approximately 150 h. In addition, the ZAB performance of Fe_SA+NC_‐NC was systematically compared with recently reported advanced catalysts, as summarized in Table S8. The Fe_SA+NC_‐NC‐based ZAB exhibits competitive overall performance in terms of open‐circuit voltage, peak power density, and cycling stability, further demonstrating its promising potential for practical ZAB applications.

## 3 Conclusion

In summary, we have successfully designed a synergistic catalyst featuring spatially coupled Fe single atoms and nanoclusters that effectively overcomes the persistent activity‐stability trade‐off in the oxygen reduction reaction. Our novel neighboring nanocluster‐induced ligand modification strategy leverages adjacent Fe nanoclusters that provide both an extra anchoring effect and modulate the electronic environment via charge transfer. This synergy of effects firmly anchors the critical OH ligand to the Fe‐N_x_ active sites, greatly enhancing its stability. This stabilized OH ligand is the key to optimizing catalytic function, as it mitigates the overly strong binding of the rate‐limiting *OH intermediate and thereby significantly lowers the reaction overpotential. Direct evidence for the favorable evolution of ORR intermediates was provided by in situ Raman spectroscopy. Consequently, the resulting Fe_SA+NC_‐NC catalyst exhibits exceptional performance with a half‐wave potential of 0.92 V and outstanding durability, retaining 95% of its initial activity after 300 h of continuous operation. Furthermore, when integrated into a practical device, the catalyst enabled a ZAB to deliver a high peak power density of 154 mW cm^−2^ and exceptional long‐term stability, outperforming the Pt/C benchmark. Ab initio molecular dynamics simulations also further verified the dynamic structural stability of these unique OH‐modified active centers under aqueous operating conditions. This work offers valuable insights and presents an effective strategy for rationally designing advanced single‐atom catalysts with simultaneously high activity and stability for energy conversion applications.

## Author Contributions

**Xue Zhao**: conceptualization, methodology, investigation, writing – original draft.**Junpeng Chen**: methodology, investigation. **Shice Wang**: methodology, validation **Zhao Yu**: methodology, Writing – review and editing. **Jing Wang**: methodology, supervision, funding acquisition. **Qiuming Peng**: methodology, validation, writing – review and editing. **Ge Li**: conceptualization, resources, supervision, writing – review and editing, methodology.

## Conflicts of Interest

The authors declare no conflicts of interest.

## Supporting information




**Supporting File**: advs77615‐sup‐0001‐SuppMat.docx.

## Data Availability

The data that support the findings of this study are available from the corresponding author upon reasonable request.
