# Supplementary material for: Nanocluster‐Assisted OH Ligand Modification Optimizes Activity and Stability of Fe‐N‐C Catalysts
Source: Adv Sci (Weinh). 2026 Sep 8:e77615. Online ahead of print. doi: 10.1002/advs.77615 (PMC13554504; doi:10.1002/advs.77615)
Supplement: Supplementary file 1 [file ADVS-9999-e77615-s001.docx]

*Supplementary Information*

**Nanocluster-Assisted OH Ligand Modification Optimizes Activity and Stability of Fe-N-C Catalysts**

*Xue Zhao, Junpeng Chen, Shice Wang, Zhao Yu, Jing Wang*, Qiuming Peng*, Ge Li**

X. Zhao, J. Chen, S. Wang, J. Wang, Q. Peng

State Key Laboratory of Metastable Materials Science and Technology, Yanshan University, Qinhuangdao, 066004, P.R. China

Corresponding Authors: Prof. Jing Wang: jwang6027@ysu.edu.cn

X. Zhao, Z. Yu, G. Li

Department of Mechanical Engineering, University of Alberta, Edmonton, Alberta, Canada

**Keywords**: oxygen reduction reaction; single atom catalysts; nanoclusters; ligand modification; DFT

**1. Methods**

**1.1 Synthesis of catalysts.** In a typical procedure, 2.97 g of 2-methylimidazole was dissolved in 100 mL of methanol to form solution A. Separately, 1.48 g of Zn(NO_3_)_2_·6H_2_O and 0.2g of Fe(acac)_3_ were dissolved in another 100 mL of methanol to form solution B. Solution A was then slowly poured into solution B under vigorous stirring at room temperature. The mixture was stirred for 24 h to form the Fe-doped ZIF-8 precursor. The resulting white precipitate was collected by centrifugation, washed three times with methanol, and dried under vacuum at 60 °C for 12 h to obtain the Fe@ZIF-8 precursor. The dried precursor was then pyrolyzed under nitrogen at 900 °C for 2 h at a ramp rate of 5 °C/min. The resulting black powder was denoted as Fe_SA+NC_-NC, which features coexisting atomically dispersed Fe sites and Fe nanoclusters embedded in a nitrogen-doped carbon matrix.

The final products were denoted as NC, Fe_SA_-NC, and Fe_SA+NP_-NC, corresponding to initial Fe(acac)_3_ amounts of 0 g, 0.1 g, and 0.4 g, respectively.

**1.2 Material characteristics.** X-ray diffraction (XRD) was performed using a SmartLab diffractometer (Rigaku, Japan) with Cu Kα radiation (λ = 1.5406 Å), covering a 2θ range of 10° to 60° at a scan rate of 4° per minute. High-resolution transmission electron microscopy (HRTEM) and scanning transmission electron microscopy (STEM) on a JEOL 2100 microscope operating at 300 kV. Inductively coupled plasma mass spectrometry (ICP-MS) was conducted using Agilent 7700/7800 systems (Agilent Technologies, USA). X-ray photoelectron spectroscopy (XPS) data were collected on an ESCALAB 250Xi instrument (Thermo Scientific, USA) with Al Kα radiation. Raman spectra were obtained from a Renishaw inVia Qontor system. X-ray absorption spectroscopy (XAS) measurements were conducted at beamline BL07A1 at the National Synchrotron Radiation Research Center (NSRRC). A Si(111) double-crystal monochromator was used to monochromatize the X-ray beam. The extended X-ray absorption fine structure (EXAFS) data were analyzed using the IFEFFIT software suite. The XANES and EXAFS data were processed with Athena software, and the χ(k) functions were Fourier transformed into R-space using a Hanning window (dk = 1.0 Å^-1^) to isolate contributions from different coordination environments. Quantitative EXAFS fitting was performed in R-space (2.0-15 Å^-1^) with k-space Fourier transforms (2.0-15 Å^-1^) using the ARTEMIS program. The fitting involved theoretical amplitude functions *F_(k)_* and phase shift functions *Φ_(k)_*, both derived from FEFF8.0 simulations.

**1.3 Electrochemical properties.** The ORR electrochemical measurements were carried out on an electrochemical workstation (CHI 760E, China) equipped with a RRDE-3A (ALS Co., Ltd, Japan) using a standard three-electrode system. The glassy carbon (GC) with a diameter of 3 mm was used as the support for the working electrode. The graphite rod and Ag/AgCl electrode were utilized as the counter electrode and reference electrode, respectively. All the potentials were calibrated to the RHE potential calculated from the following equation: $E\left( vs.RHE \right)=E\left( vs.Ag/AgCl \right)+0.198 V+0.059\times pH$

 (7)

To prepare the catalyst ink, 5 mg of catalyst powder was dispersed in a solvent mixture consisting of 200 μL isopropanol, 800 μL deionized water, and 20 μL of 5 wt% Nafion solution. The mixture was ultrasonicated for 30 minutes to form a uniform suspension. The ink was then drop-cast onto the glassy carbon disk with a final catalyst loading of 0.6 mg cm^-2^, followed by air-drying at room temperature to obtain a uniform thin film.

Prior to electrochemical measurements, the electrolyte was saturated with either O_2_ or Ar by bubbling the gas through the solution for at least 30 minutes. Linear sweep voltammetry (LSV) was conducted in O_2_-saturated 0.1 M KOH at a scan rate of 10 mV s^-1^ at various rotation speeds (400-2000 rpm) to assess the catalytic activity. ORR polarization curves were corrected for background by subtracting those obtained under Ar-saturated conditions. For cyclic voltammetry (CV), measurements were performed at 50 mV s^-1^ between 0 V and 1.2 V.

The number of electrons transferred (n) and the kinetic current density (j_k_) were determined from Koutecky-Levich (K-L) analysis according to the following relation:

$\frac{1}{j}=\frac{1}{j_{L}}+\frac{1}{j_{K}}=\frac{1}{B\omega^{1/2}}+\frac{1}{j_{K}}$ (8)

$B=0.62nFD_{0}^{2/3}v^{-1/6}C_{0}$ (9)

Here, *j* is the measured current density, *ω* is the angular rotation speed, *D_O_* is the diffusion coefficient of O_2_ (1.9 × 10^-5^ cm^2^ s^-1^), *C_O_* is the concentration of dissolved O_2_ (1.2 × 10^-6^ mol cm^-3^), and *ν* is the kinematic viscosity of the electrolyte (0.01 cm^2^ s^-1^). The hydrogen peroxide yield (H_2_O_2_%) and electron transfer number (*n*) were also calculated from the ring and disk currents at 1600 rpm using the following equations:

$H_{2}O_{2}(\%)=200\times\frac{\frac{I_{r}}{N}}{I_{d}+\frac{I_{r}}{N}}$ (10)

$n=4\times\frac{I_{d}}{I_{d}+\frac{I_{r}}{N}}$ (11)

where *I_d_* and *I_r_* are the disk and ring currents, and *N* is the collection efficiency of the ring electrode.

**1.4 Computational method.** All of the calculations were performed using the projector-augmented wave method as implemented in the Vienna *ab initio* simulation package (VASP)^[1]^. The exchange-correlation potentials were treated in the generalized gradient approximation by the Perdew-Burke-Ernzerhof form^[2]^. The energy cutoff of 500 eV was set for the plane-wave expansion. All of the structural relaxations were performed until the Hellmann-Feynman forces were <10^−5^ eV/Å. Brillouin zone with a Gaussian broadening of 0.05 eV was performed. The centered Monkhorst-Pack k-point meshes for sampling the Brillouin zone was 5 × 5 × 1. The van der Waals (vdW) interaction was involved via the semi-empirical DFT-D2 field method^[3]^. The Gibbs free energy was calculated as follows:

ΔG = ΔE + ΔZPE − TΔS,

where ΔE was the DFT calculated adsorption energy difference from the initial to the final states. ΔZPE was the change in zero-point energy (ZPE). T was fixed at 289.15 K in our study and ΔS was the entropy change. The nature of the stationary point and ZPE corrections was characterized by calculating the harmonic vibration frequency. The Gibbs free energy change (ΔG_i_, I = 1–4) for each elementary step can be summarized as follows:

ΔG_1_ = 4.92 − ΔG_∗OOH_

ΔG_2_ = ΔG_∗OOH_ − ΔG_∗O_

ΔG_3_ = ΔG_∗O_ − ΔG_∗OH_

ΔG_4_ = ΔG_∗OH_

The theoretical overpotential η^ORR^ for a given electrocatalyst can be evaluated as

η^ORR^ = 1.23 V − min{ΔG_1_, ΔG_2_, ΔG_3_, ΔG_4_}/e.

**1.5 In-situ Raman spectroscopic tests.** I*n-situ* Raman spectra were recorded with a Renishaw InVIa Raman Microscope using a 532 nm line of an Ar-ion laser, and the microscope objective lens was ×50 magnification. Before the experiment, the Raman frequency was calibrated using a Si wafer (520.6 cm⁻¹). The *in-situ* spectro-electrochemical cell was also carried out with a three-electrode configuration. During the ORR process, the cell was controlled by an electrochemical workstation (CHI760E) for the *i-t* tests from 1.0 to 0.5 V vs. RHE. Each potential was held for 10 min, while the Raman spectra were collected simultaneously for 8 min with 20 accumulations to enhance its signal-to-noise ratio.

**1.6 Quasi-operando XPS tests.** The catalyst ink was prepared by dispersing the catalyst powder with a Nafion binder in ethanol, followed by ultrasonication for 30 min to obtain a homogeneous suspension. The resulting ink was drop cast onto carbon paper and dried at room temperature for 12 h. Electrochemical polarization was conducted in a three electrode configuration using 0.1 M KOH electrolyte. The catalyst coated carbon paper served as the working electrode. Chronoamperometry was performed at selected potentials between 0.9 and 0.5 V vs. RHE, and each polarization was maintained for 30 min. After polarization, the working electrode was removed from the electrolyte, thoroughly rinsed with deionized water and then with ethanol to remove residual electrolyte, and dried at room temperature for 12 h. The dried electrodes were subsequently analyzed by X ray photoelectron spectroscopy to obtain quasi operando surface chemical states corresponding to the applied electrochemical potentials.

**1.7 ZAB performance.** The temperature was controlled by a programmable box (GDJS-100, Beijing YaShiLin Testing Equipment Co., Ltd., China). 6 M KOH with 0.2 M Zn(CH_3_COOH)_2_ was the electrolyte, while the polished zinc plate was used as the anode. The air cathode was made by drop casting the catalyst ink which consists of the catalysts, ionomer (Nafion solution, 5 wt %), isopropanol and H_2_O onto the hydrophilic carbon paper with a mass loading of 1.0 mg cm^-2^. The polarization curves were obtained by using the CHI760E electrochemical workstation, and the galvanostatic charge-discharge tests were performed on a battery measurement system (Land CT2001A, China).

**2. Results and Discussion**

**Figure S1.** The overall SEM morphologies of the NC (a), Fe_SA_-NC (b), Fe_SA+NC_-NC (c) and Fe_SA+NP_-NC (d).

**Figure S2.** N_2_ adsorption/desorption plots of the NC, Fe_SA_-NC, Fe_SA+NC_-NC and Fe_SA+NP_-NC.

**Figure S3.** The structural characteristics of the Fe_SA_-NC. a) TEM images. b) AC-HAADF-STEM image. c) EDS elemental mapping.

**Figure S4.** The structural characteristics of the Fe_SA+NP_-NC. a) TEM images. b) AC-HAADF-STEM image. c) EDS elemental mapping.

**Figure S5.** High-resolution XPS spectra of C 1s for Fe_SA_-NC and Fe_SA+NP_-NC

**Figure S6.** High-resolution XPS spectra of O 1s for catalysts.

**Figure S7.** CV curves of the NC (a), Fe_SA_-NC (b), Fe_SA+NC_-NC (c) and Fe_SA+NP_-NC (d) in a 0.1 M KOH solution within a potential range from 0 to 1.2 V (scan rate: 50 mV s^–1^).

**Figure S8.** CV curves of the NC (a), Fe_SA_-NC (b), Fe_SA+NC_-NC (c) and Fe_SA+NP_-NC (d) with different rotation rates during the RDE test.

**Figure S9.** Kinetic current density j_k_@ 0.85 V for NC, Fe_SA_-NC, Fe_SA+NC_-NC, Fe_SA+NP_-NC, and Pt/C under ORR testing.

**Figure S10.** CV curves of the NC (a), Fe_SA_-NC (b), Fe_SA+NC_-NC (c) and Fe_SA+NP_-NC (d) for ORR at various scan rates (5, 10, 15, 20, 25 and 30 mV s^-1^ ) from 1.1 to 1.2 V vs. RHE in 0.1 M KOH solution.

**Figure S11.** (a) Nyquist plots recorded at various potentials ranging from 1.0 V to 0.6 V during the discharge and charge processes. (b) The corresponding distribution of relaxation times analysis derived from the EIS data, revealing the evolution of time constants ($\tau$) at different applied potentials.

**Figure S12.** Potential-resolved in situ Raman intensity map collected from 1.0 to 0.5 V vs. RHE of Fe_SA_-NC.

**Figure S13.** Quasi-operando XPS spectra at different potentials of Fe_SA_-NC. (a) Fe 2p. (b) O 1s.

**Figure S14.** Raman spectra before and after cycling of Fe_SA+NC_-NC.

|  | * | *O_2_ | *OOH | *O | *OH |
| --- | --- | --- | --- | --- | --- |
| FeN_4_/Fe_3_N_6_(I) |    |  |  |  |  |
| FeN_4_/Fe_4_N_6_(I)  (FeN_4_ side) |  |  |  |  |  |
| FeN_4_/Fe_4_N_6_(I)  (Fe_4_N_6_ side) |    |    |    |    |    |
| FeN_4_/Fe_5_N_6_(I) |  |  |  |  |  |
| FeN_4_/Fe_3_N_6_(II) |  |  |  |  |  |
| FeN_4_/Fe_4_N_6_(II) |  |  |  |  |  |
| FeN_4_/Fe_5_N_6_(II) |  |  |  |  |  |

**Figure S15.** Structures of key oxygen-containing intermediates for the ORR on Fe_SA_/Fe_NC_ catalysts; silver, brown, red, white, and orange spheres represent N, C, O, H, and Fe, respectively.

|  | * | *O_2_ | *OOH | *O | *OH |
| --- | --- | --- | --- | --- | --- |
| FeN_4_-OH/ Fe_3_N_6_(I) |  |  |  |  |  |
| FeN_4_-OH/ Fe_4_N_6_(I) |  |  |  |  |  |
| FeN_4_-OH/ Fe_5_N_6_(I) |  |  |  |  |  |
| FeN_4_-OH/ Fe_3_N_6_(II) |  |  |  |  |  |
| FeN_4_-OH/ Fe_4_N_6_(II) |  |  |  |  |  |
| FeN_4_-OH/ Fe_5_N_6_(II) |  |  |  |  |  |

**Figure S16.** Structures of key oxygenated intermediates along the 4e⁻ ORR on Fe_SA_/Fe_NC_ catalysts with the Fe_SA_ site hydroxyl-modified.

|  | * | *O_2_ | *OOH | *O | *OH |
| --- | --- | --- | --- | --- | --- |
| FeN_4_/ Fe_3_N_6_-OH(I) |  |  |  |  |  |
| FeN_4_-OH/ Fe_3_N_6_-OH(I) |  |  |  |  |  |
| FeN_4_/ Fe_4_N_6_-OH(I) |  |  |  |  |  |
| FeN_4_-OH/ Fe_4_N_6_-OH(I) |  |  |  |  |  |
| FeN_4_/ Fe_5_N_6_-OH(I) |  |  |  |  |  |
| FeN_4_-OH/ Fe_5_N_6_-OH(I) |  |  |  |  |  |

**Figure S17.** Structures of key oxygenated intermediates along the 4e⁻ ORR on Fe_SA_/Fe_NC_-OH(I) and Fe_SA_-OH/Fe_NC_-OH(I) catalysts.

**Figure S18.** Free-energy diagrams for 4e⁻ ORR on Fe_SA_/Fe_NC_ catalysts; blue dashed lines denote the rate-determining step for ORR.

**Figure S19.** Free-energy diagrams for 4e⁻ ORR on Fe_SA_–OH/Fe_NC_ catalysts

**Figure S20.** Free-energy diagrams for 4e⁻ ORR on Fe_SA_/Fe_AC_-OH(I) and Fe_SA_-OH/Fe_AC_-OH(I).

**Figure S21.** Fe 3d projected density of states of the Fe active site in the (a) FeN_4_, (b) Fe_5_N_6_, and (c) Fe_5_N_6_-OH models. The dashed vertical line represents the Fermi level.

**Figure S22.** Long-term cycling stability of ZAB based on Fe_SA+NC_-NC and 20 % Pt/C+RuO_2_ at 10 mA cm^-2^.

**Table S1.** Chemical composition of the catalysts recorded by XPS.

|  | C (at%) | N (at%) | O (at%) | Fe (at%) |
| --- | --- | --- | --- | --- |
| Fe_SA_-NC | 91.56 | 3.14 | 5.11 | 0.19 |
| Fe_SA+NC_-NC | 90.95 | 3.95 | 4.83 | 0.27 |
| Fe_SA+NP_-NC | 89.75 | 4.29 | 5.52 | 0.44 |

**Table S2.** Metal concentration in the different samples determined by inductively coupled plasma optical emission spectroscopy (ICP-OES).

| Samples | Fe concentration (wt.%) |
| --- | --- |
| Fe_SA_-NC | 0.57 |
| Fe_SA+NC_-NC | 1.21 |
| Fe_SA+NP_-NC | 1.96 |

**Table S3.** Comparison of ORR performance between the Fe_SA+NC_-NC and other non-precious catalysts reported in the literatures under O_2_-saturated 0.1 M KOH solutions.

|  | Electrocatalysts | E_(onset)_  ( V vs.RHE) | E_(1/2)_  ( V vs.RHE) | Ref. |
| --- | --- | --- | --- | --- |
| 1 | Fe_SA+NC_-NC | 1.050 | 0.920 | This work |
| 2 | Fe_SA+NP_-NC | 0.966 | 0.890 | This work |
| 3 | Fe_SA_-NC | 0.949 | 0.860 | This work |
| 4 | FeN_4_SeC_2_ | 0.975 | 0.875 | ^[4]^ |
| 5 | Fe_SA_/Fe_AC_@PPy/CC | 0.970 | 0.830 | ^[5]^ |
| 6 | Zn-B/N-C | 0.960 | 0.886 | ^[6]^ |
| 7 | FeNi-S/NB/C | 0.920 | 0.845 | ^[7]^ |
| 8 | FeCo-NPC | 0.960 | 0.830 | ^[8]^ |
| 9 | La-Cl SAs/NHPC | 0.975 | 0.910 | ^[9]^ |
| 10 | Ce SAs/PSNC | 0.975 | 0.900 | ^[10]^ |
| 11 | Co@DMOF-900 | 0.930 | 0.866 | ^[11]^ |
| 12 | YN_4_-Cl-2 | 0.91 | 0.850 | ^[12]^ |
| 13 | Mn-SAS | 0.990 | 0.850 | ^[13]^ |
| 14 | ZnCoFe-TAC/SNC | 0.963 | 0.901 | ^[14]^ |

**Table S4.** Fe contents after CV cycling determined by ICP-MS.

| Sample | Fe concentration |
| --- | --- |
| Electrolyte (KOH) | 0 (μg/L) |
| Fe_SA+NC_-NC (Titanium mesh) | 1.18 (wt.%) |

**Table S5.** EXAFS fitting parameters at the Fe k-edge for various samples (*Ѕ*_0_^2^=0.94).

| Sample | Path | | | *CN^a^* | *R*(Å)*^b^* | *σ*^2^ (Å^2^)*^c^* | | Δ*E*_0_(eV)*^d^* | *R* factor |
| --- | --- | --- | --- | --- | --- | --- | --- | --- | --- |
| Fe K-edge (*Ѕ*_0_^2^=0.90) | | | | | | | | | |
| Fe_SA+NC_-NC | | Fe-N | 4.0±0.1 | | 2.00±0.01 | | 0.007 | 2.3 | 0.0022 |
|  |  | Fe-N-Fe | 0.4±0.1 | | 3.02±0.02 | | 0.003 | 4.4 |  |
| After  Cycles | | Fe-O | 1.0±0.1 | | 1.85±0.01 | | 0.007 | -0.5 | 0.0004 |
|  |  | Fe-N | 4.0±0.1 | | 2.00±0.01 | | 0.007 |  |  |
|  |  | Fe-N/O-Fe | 0.4±0.1 | | 3.00±0.01 | | 0.004 | 2.6 |  |

**Table S6.** Fe_SA_/Fe_NC_ catalysts before and after hydroxylation showing the Fe-O bond length d_Fe-O_ and the O–O bond length d_O-O_ for the *O₂ intermediate, together with the O_2_ adsorption energy Δ*E_ads_*_._

| catalysts | *d*_Fe-O_ ***O_2_ (Å) | *d*_O2_ (Å) | Δ*E*_ads_ (eV) |
| --- | --- | --- | --- |
| FeN_4_/Fe_3_N_6_(I) | 1.79 | 1.30 | -1.20 |
| FeN_4_-OH/Fe_3_N_6_(I) | 1.92 | 1.31 | -0.97 |
| FeN_4_/Fe_4_N_6_(I) | 1.79 | 1.30 | -1.43 |
| FeN_4_-OH/Fe_4_N_6_(I) | 1.93 | 1.31 | -1.12 |
| FeN_4_/Fe_5_N_6_(I) | 1.78 | 1.30 | -1.27 |
| FeN_4_-OH/Fe_5_N_6_(I) | 1.91 | 1.31 | -0.90 |
| FeN_4_/Fe_3_N_6_(II) | 1.77 | 1.30 | -1.23 |
| FeN_4_-OH/Fe_3_N_6_(II) | 1.91 | 1.30 | -0.70 |
| FeN_4_/Fe_4_N_6_(II) | 1.78 | 1.30 | -1.23 |
| FeN_4_-OH/Fe_4_N_6_(II) | 2.02 | 1.30 | -0.74 |
| FeN_4_/Fe_5_N_6_(II) | 1.77 | 1.30 | -1.24 |
| FeN_4_-OH/Fe_5_N_6_(II) | 1.97 | 1.30 | -0.56 |

**Table S7.** The binding energy of OH ligands on Fe sites with/without adjacent clusters.

| Sample | Binding energy (eV) |
| --- | --- |
| Fe_SA_-NC | 0.44 |
| Fe_SA+NC_-NC | 0.40 |

**Table S8**. Comparison of ZAB performance of Fe_SA+NC_-NC and other reported advanced catalysts

| Catalysts | Power density/ mW cm^-2^ | Open circuit/V | Cycle time | Reference |
| --- | --- | --- | --- | --- |
| Fe_SA+NC_-NC | 154 | 1.487 | 300 h@10 mA cm^-2^ | **This Work** |
| Co_9_S_8_/Fe_7_S_8_@NSCNTs | 118.4 | 1.44 | 207 h@10 mA cm^-2^ | *Adv. Energy Mater*., **2025**, 15, e03189 |
| Ir_SA_-MnO_x_ | 130 | 1.49 | 700 h@1 mA cm^-2^ | *Adv. Mater*., **2025**, 37, 2412950 |
| CCSO/NC-2 | 104.8 | 1.43 | 300 h@10 mA cm^-2^ | *Adv. Mater*., **2023**, 35, 2303488 |
| Co-CoN_4_@NCNs | 119 | 1.47 | 500 h@10 mA cm^-2^ | *Adv. Funct. Mater*., **2022**, 32, 2207331 |
| Co_AC_-Mo_x_C/CC | 146.4 | 1.49 | 300 h@10 mA cm^-2^ | *Adv. Funct. Mater*., **2025**, 35, 2506077 |
| Fe−Se/NC | 135 | 1.47 | 200 h@20 mA cm^-2^ | *Angew. Chem. Int. Ed*., **2023**, 62, e202219191 |
| PdMo | 154 | 1.48 | 116.6 h@10 mA cm^-2^ | *Nature*, **2019**, 574, 81-85 |
| FeCo-N_3_O_3_@C | 143 | 1.43 | 220 h@10 mA cm^-2^ | *Nat. Synth.*, **2024**, 3, 878-890 |
| CR-Co/ClNC | 176.6 | 1.50 | 30 h@10 mA cm^-2^ | *Nat. Commun*., **2024**, 15, 1675 |
| NDGs-800 | 115.2 | 1.45 | 78 h@10 mA cm^-2^ | *ACS Energy Lett.*, **2018**, 3, 1183 |
| Co–Co_2_Nb_5_O_14_ | 114.07 | 1.496 | 666 h@10 mA cm^-2^ | *Adv. Sci*. **2025**, 12, 2413796 |
| Fe–N–C/Fe_3_C-op | 137.4 | 1.568 | 400 h@5 mA cm^-2^ | *Adv. Sci.*, **2023**, 10, 2301656 |

**References**

[1]	aP. E. J. P. r. B. Blöchl, *Phys. Rev. B* **1994**, *50*, 17953; bG. Kresse, J. Furthmüller, *Phys. Rev. B* **1996**, *54*, 11169.

[2]	aJ. P. Perdew, K. Burke, M. Ernzerhof, *Phys. Rev. Lett.* **1996**, *77*, 3865; bP. Hohenberg, W. Kohn, *Phys. Rev. B* **1964**, *136*, B864; cW. Kohn, L. J. Sham, *Phys. Rev. B* **1965**, *140*, A1133.

[3]	E. Jung, H. Shin, B.-H. Lee, V. Efremov, S. Lee, H. S. Lee, J. Kim, W. Hooch Antink, S. Park, K.-S. Lee, S.-P. Cho, J. S. Yoo, Y.-E. Sung, T. Hyeon, *Nat. Mater.* **2020**, *19*, 436-442.

[4]	Y. Cao, Y. Liu, X. Zheng, J. Yang, H. Wang, J. Zhang, X. Han, Y. Deng, G. Rupprechter, W. Hu, *Angew. Chem. Int. Ed.* **2025**, *64*, e202423556.

[5]	Q. Liu, P. Qiao, D. Shen, Y. Xie, B. Wang, T. Han, H. Shi, L. Wang, H. Fu, *Energy Environ. Sci* **2025**, *18*, 2839-2851.

[6]	J. Wang, H. Li, S. Liu, Y. Hu, J. Zhang, M. Xia, Y. Hou, J. Tse, J. Zhang, Y. Zhao, *Angew. Chem. Int. Ed.* **2021**, *133*, 183-187.

[7]	K. Yu, J. Qin, H. Zhang, S. Zhang, Y. Cao, C. Xie, H. Yang, W. Xiao, W. Li, G. Zhang, Y. Luo, G. Cao, J. Wang, X. Li, *Adv. Energy Mater.* **2025**, 2501091.

[8]	Z. Pei, H. Zhang, Y. Guo, D. Luan, X. Gu, X. W. Lou, *Adv. Mater.* **2024**, *36*, 2306047.

[9]	L. Yin, M. Sun, S. Zhang, Y. Huang, B. Huang, Y. Du, *Adv. Mater.* **2025**, *37*, 2416387.

[10]	L. Yin, S. Zhang, M. Sun, S. Wang, B. Huang, Y. Du, *Adv. Mater.* **2023**, *35*, 2302485.

[11]	S. Yuan, J. Zhang, L. Hu, J. Li, S. Li, Y. Gao, Q. Zhang, L. Gu, W. Yang, X. Feng, B. Wang, *Angew. Chem. Int. Ed.* **2021**, *60*, 21685-21690.

[12]	D. Liu, X. Wan, J. J. S. Shui, *Small* **2024**, *20*, 2406078.

[13]	M. Tong, F. Sun, G. Xing, C. Tian, L. Wang, H. Fu, *Angew. Chem. Int. Ed.* **2023**, *62*, e202314933.

[14]	C. Chen, J. Chai, M. Sun, T. Guo, J. Lin, Y. Zhou, Z. Sun, F. Zhang, L. Zhang, W. Chen, L. Yujing, *Energy Environ. Sci* **2024**, *17*, 2298-2308.
